# Adenoviral vector vaccine platforms in the SARS-CoV-2 pandemic

**DOI:** 10.1038/s41541-021-00356-x

**Published:** 2021-08-05

**Authors:** Samir Andrade Mendonça, Reka Lorincz, Paul Boucher, David T. Curiel

**Affiliations:** Washington University in Saint Louis, School of Medicine, Biologic Therapeutics Center, Radiation Oncology Department. 660 South Euclid Avenue, St. Louis, MO USA

**Keywords:** Vaccines, Biotechnology

## Abstract

Adenoviral vectors have been explored as vaccine agents for a range of infectious diseases, and their ability to induce a potent and balanced immune response made them logical candidates to apply to the COVID-19 pandemic. The unique molecular characteristics of these vectors enabled the rapid development of vaccines with advanced designs capable of overcoming the biological challenges faced by early adenoviral vector systems. These successes and the urgency of the COVID-19 situation have resulted in a flurry of candidate adenoviral vector vaccines for COVID-19 from both academia and industry. These vaccines represent some of the lead candidates currently supported by Operation Warp Speed and other government agencies for rapid translational development. This review details adenoviral vector COVID-19 vaccines currently in human clinical trials and provides an overview of the new technologies employed in their design. As these vaccines have formed a cornerstone of the COVID-19 global vaccination campaign, this review provides a full consideration of the impact and development of this emerging platform.

## Introduction

In late 2019 the first patients infected by the severe acute respiratory syndrome coronavirus 2 (SARS-CoV-2) were found in Wuhan, China. One year later over 157 million cases have been reported worldwide, causing 3.2 million fatalities, and having an unprecedented impact on the world economy^1–3^ (WHO COVID-19 Weekly Epidemiological Update, as of 11 May 2021). Additionally, persistent uncontrolled circulation of the virus has raised concerns about the emergence of new variants with enhanced transmissibility, increased disease severity, or vaccine escape potential. Of note, the Center for Disease Control and Prevention (CDC) has classified three variants of major concern due to their potentially increased infectivity: B.1.1.7 (first identified in the UK), 501Y.V2 (first identified in South Africa), and P.1 (first identified in Brazil)^4^. Although improvements in the clinical management of hospitalized patients have led to decreased mortality rates^5^ and the combination of social distancing and masking has mitigated transmission, a more effective long-term strategic approach is necessary: a vaccine capable of eliciting a strong long-term immune response that can be manufactured at a global scale.

The global demand for effective vaccines has triggered a race in which a wide range of vaccine platforms have been assessed; from the early stages of research through phase 3 clinical trials. These vaccines include traditional technologies, such as live-attenuated and inactivated viruses, as well as protein subunit vaccines^6,7^. However, the urgency of the COVID-19 pandemic has resulted in a flurry of development in viral vector and mRNA vaccines. These approaches rely on the delivery of either DNA or mRNA encoding antigens to induce an immune response against the pathogen. This carries a distinct advantage over other technologies - the ability to rapidly develop a novel vaccine by simply altering the delivered nucleic acid sequence. This has resulted in viral vector-based and mRNA vaccines obtaining emergency use authorizations more rapidly than any other vaccine technology, with seven out of the fourteen vaccines currently approved for at least emergency or limited use globally being adenoviral vector or mRNA based^8^. In this review, we describe the biological features of adenoviral (Ad) vectors that position them as potential frontrunners in contrast with other technologies, particularly mRNA vaccines, and detail the current Ad candidates for a SARS-CoV-2 vaccine.

## mRNA vaccines

The concept of mRNA vaccines was initially developed in the 1990s^9,10^. The basis of this technology is the delivery of mRNA encoding an antigen from a target pathogen to the host’s cells. These cells then produce the antigen, which the immune system recognizes as foreign, resulting in an immune response and potentially successful development of immunity. Advances in mRNA production and gene delivery have driven a surge of groups interested in the technology for numerous applications, including cancer, inherited diseases, and vaccines^11^. The candidate mRNA is typically delivered in a lipid nanoparticle (LNP), increasing its stability in vivo and ability to successfully enter the host’s cells and be delivered to the cytosol. Advances in nanotechnology have resulted in the development of efficient carriers which are generally nontoxic, and ideally nonimmunogenic, allowing for repeated dosing of the LNP-mRNA system^12^.

These advances have enabled the first major clinical success for mRNA: the approval of COVID-19 vaccines. In late 2020, mRNA vaccines developed by Pfizer-BioNTech and Moderna were approved for emergency use in multiple countries. As of April 2021, in the United States alone, over 78 million people had received at least one dose of the Pfizer-BioNTech vaccine, and over 71 million people had received the Moderna vaccine^13^. This breakneck pace of vaccine development, testing, and approval represents a stunning achievement previously impossible, strongly demonstrating the utility of mRNA vaccines in emerging pathogen outbreaks. The ease and rapidity of assembling new mRNA sequences into existing vaccine formulations will undoubtedly position mRNA vaccines as vaccine forerunners in the future as well. Unsurprisingly, Pfizer-BioNTech and Moderna appear to be leading the charge against new SARS-CoV-2 variants with their mRNA technologies, with both companies having announced studies to develop booster vaccines^14,15^. Encouragingly, recent data from Qatar also suggests variant-specific boosters may not be necessary, where the Pfizer-BioNTech vaccine was shown to be effective against two variants of concern^16^.

Despite this promise, lingering biological and practical challenges remain with mRNA vaccines. Although rare, severe anaphylactic reactions have been reported with the Pfizer-BioNTech and Moderna vaccines^17^. At the time of writing the source of these reactions remains unclear but has been hypothesized to be the polyethylene glycol (PEG) in the lipid nanoparticle used to deliver the mRNA^17,18^. New screening contraindications for allergic reactions to vaccine components such as PEG or polysorbate 80 have helped manage this issue^19^. Developing an immune response to the delivery system may undercut a key advantage of mRNA vaccines-their “plug and play” nature whereby a new vaccine could be developed simply by altering the delivered mRNA sequence. It remains to be seen whether this will remain an issue impacting only a small number of people, or if new delivery systems will need to be developed, or if the reaction is caused by another vaccine component entirely.

Additionally, while the humoral immune response to mRNA vaccines has been well characterized, the cellular immune response is less well understood, and has been characterized as generating a relatively weak CD8^+^ T cell response, which may be important in generating strong long-term immunity^20^. Initial studies reported primarily CD4^+^ T cell priming by mRNA vaccines, but one recent study indicated robust CD8^+^ T cell responses as well as CD4^+^ T cell responses in both SARS-CoV-2 naïve and recovered individuals that were vaccinated. In this study, a prime-boost regimen was used (two sequential temporally separated vaccine doses), and the gradual development of antigen-specific CD8^+^ T cells observed highlights the importance of such a dosing protocol. Studies are still ongoing to determine the longevity of mRNA vaccine-induced memory T cell responses, which are typically exceptionally durable in other vaccine settings^21^.

On the practical front, challenges producing and distributing mRNA vaccines have limited them to developed countries with established vaccine infrastructure. The in vitro reaction to produce mRNA relies on multiple costly GMP grade products, resulting in mRNA vaccines being priced significantly higher than Ad vector vaccines^22^. Additionally, the Pfizer-BioNTech vaccine must be stored long-term at −70 °C, while the Moderna vaccine must be stored at −20 °C. Although each vaccine can tolerate storage at 2–8 °C for 5 and 30 days respectively, these stringent long-term storage requirements will present distribution challenges, especially in areas without existing cold-chain infrastructure. In contrast, leading Ad vaccines can be stored three to six times longer than Moderna’s mRNA vaccine at 2–8 °C^23^. However, Pfizer-BioNTech recently submitted data to the Food and Drug Administration (FDA) to update the storage requirements to a more reasonable −25 to −15 °C, and Moderna has initiated a trial with a vaccine that may be refrigerator stable^24,25^. In parallel, an mRNA-based vaccine under clinical investigation in China (ARCoV; ChiCTR2000034112) was shown to be stable at room temperature for at least 7 days^26^. These developments highlight the possibility that the cold-storage issues with mRNA vaccines may be solved through further research.

mRNA has undoubtedly proven itself a promising technology for the rapid development of effective vaccines against emerging infectious diseases. However, additional studies to reduce the side effects and cost of these vaccines need to be undertaken before they can be considered a definitive solution for the billions of people affected by the SARS-CoV-2 pandemic, as well as future disease outbreaks.

## Adenoviral vector vaccines

In addition to mRNA vaccines, the most advanced technology for COVID-19 vaccines is Ad vectors^8^. Ads are non-enveloped double-stranded DNA viruses most commonly responsible for mild self-limiting respiratory and ocular infections in humans^27^. Over 150 primate Ads have been characterized, with many Ads in development for vaccine purposes^28,29^. Like mRNA vaccines, Ad vaccines are a relatively new technology, although Ads have been used as gene delivery vehicles since the earliest days of gene therapy. To generate a vector, the E1 and/or E3 viral genes enabling replication (discussed further in the following sections) are deleted and replaced with the transgene of interest - such as an antigen. This renders the virus incapable of producing further copies of its genome after delivery, instead of producing the antigen of interest. Ads possess several advantages over mRNA vaccines, including the previously mentioned cost and thermostability^23^. Additionally, the flexible viral biology affords the opportunity to engineer vectors with increased vaccine efficacy-for a comprehensive review of the immunogenicity of Ad vector vaccines, see^30^.

Ad vectors also have several features that position them as ideal vaccine candidates in comparison to other viral vectors. A key aspect of vector safety is the fate of the viral genome after delivery. Some viral vectors such as those based on lentiviruses are known to integrate into the host genome, potentially resulting in genotoxicity^31^. Such vectors are unsuitable for a widespread vaccination campaign where patient follow-up is impossible, and their utility has mostly been limited to ex vivo cell therapies^32^. In contrast, Ads have not been reported to significantly integrate into the host genome, with the viral backbone remaining episomal^27^. Additionally, the deletion of E1/E3 replication genes results in high packaging capacity allowing the incorporation of large transgene sequences^33,34^.

Importantly, the broad tissue tropism of Ads and their ability to drive strong expression of the target antigen helps position them as one of the most immunogenic viral vectors^35–37^. This capacity to elicit strong immune responses has been exploited to develop vaccine candidates for infectious diseases such as Acquired immunodeficiency syndrome (AIDS), Ebola virus disease, Zika virus disease, Malaria and Tuberculosis^38–42^ and for cancer immunotherapies^43^. Among these candidates, a recombinant Ad serotype 26 vaccine expressing a Zaire Ebola virus glycoprotein has recently demonstrated the ability to protect humans against Ebola virus disease^44^.

Finally, Ads can be readily scaled to meet the global vaccine demand; Janssen/Johnson & Johnson are reported to be planning to produce one billion doses of their COVID-19 vaccine^45^. Ad vectors can be easily grown in 20 L bioreactors with yields resulting in sufficient vaccine doses for 15,000 patients, assuming 2 doses are required to achieve vaccine efficacy and considering the expected loss of virus particles during downstream processing and quality analysis^46^. Scale up to a 500 L bioreactor has been reported, demonstrating the potential for easily generating large scale batches of Ad vectors^47^. Additionally, many viral vaccine manufacturers already have a well-established manufacturing platform for Ads providing a proven and affordable vaccine solution.

Despite the favorable scientific, clinical, and practical features of the numerous Ad vectors, several considerations must be appreciated with respect to these vaccine candidates, especially regarding pre-existing anti-Ad immunity and potential adverse events. Table 1 summarizes all the Ad vector vaccines currently in clinical trials as well as their technical features.

**Table 1:** Adenovirus based vaccine candidates for SARS-CoV-2 immunization currently in clinical trials and their main technical features.

Developer	Name of candidate vaccine	Adenovirus species serotype	SARS-CoV-2 antigen payload	Most advanced stage of clinical trial	Location of development	Emergency use	Approved	Use suspension
CanSino Biologics Inc. and Beijing Institute of Biotechnology	Ad5-nCOV	Human; Ad5	Full-length Spike protein	Phase 3	China	Yes	China	No
ImmunityBio, Inc. and NantKwest Inc.	hAd5-S-Fusion+N-ETSD	Human; Ad5	Spike protein fused to the nucleocapsid protein	Phase 1	USA	No	No	NA
Vaxart	VXA-COV2-1	Human; Ad5	Full-length spike protein	Phase 1	USA	No	No	NA
Altimmune, Inc.	AdCOVID	Human; Ad5	RBD domain of spike protein	Phase 1	USA	No	No	NA
Janssen Vaccines & Prevention B.V. (Johnson & Johnson)	Ad26.COV2-S	Human, Ad26	Pre-fusion stabilized spike protein	Phase 3	Europe	Yes	No	NA
Gamaleya Research Institute	Gam-COVID-Vac/ SputnikV	Human; Ad5 and Ad26	Full-length spike protein	Phase 3	Russia	Yes	Yes	
ReiThera/LEUKOCARE/Univercells	GRAd-COV2	Gorilla; GRAd32	Pre-fusion stabilized spike protein	Phase 1	Italy	No	No	NA
University of Oxford/ AstraZeneca	ChAdOX1-nCoV	Chimpanzee; ChAdY25	Full-length spike protein	Phase 3	UK	Yes	Yes	Denmark
Washington University in Saint Louis/Bharat Biotech International Limited	ChAd-SARS-CoV-2/ BBV154	Chimpanzee; Ad36	Pre-fusion stabilized spike protein	Phase 1	India	No	No	NA
Gritstone Oncology	ChAdV68-S and ChAdV68-S-TCE	Chimpanzee; Ad68	Spike protein alone or associated with SARS-CoV-2 T cell epitope	Phase 1	USA	No	No	No

The DRAFT landscape of COVID-19 candidate vaccines-May 2021.

## Engineering the adenovirus genome

Engineering of the Ad genome has played a critical role in developing vectors for vaccines. The genome consists of around 40 kb of linear dsDNA, with the exact size varying by serotype. It contains genes encoding at least 50 viral proteins, a packaging signal, and two flanking inverted terminal repeats (ITRs)^48^. These genes can be classified on the order of transcription during the wild type Ad infection cycle: the early genes (E 1–4) that encode proteins that initiate and sustain the molecular events of the viral DNA replication, and the late genes (L 1–5) that encode structural proteins such as the fiber, hexon and penton base^49–51^.

The E1 genes encode two transcripts, E1A and E1B, that encode proteins that act together to render the host cell more susceptible to viral replication. E1A proteins stimulate host cell entry into the S phase, while E1B proteins act to hamper the p53-mediated apoptosis that would ordinarily take place in response to the changes generated by the E1A proteins^49^. Critically, these genes also encode transcription factors necessary to continue viral gene expression, and deletion of E1A and E1B is therefore sufficient to render virus replication-incompetent^51^.

Moving forward, the E2 gene encodes the viral DNA polymerase, the DNA binding protein (DBP), and the Ad preterminal protein (dTP) are transcribed, which act together to replicate the virus genome^49,50,52^. The E3 gene encodes proteins that act in host immune modulation, hampering the capacity of the infected host cells to trigger immune responses that would lead to its elimination^49,50^. Finally, the E4 transcripts encode for at least 6 different open reading frames that have distinct roles during viral replication: gene expression regulation, viral mRNA metabolism, viral DNA replication, and apoptosis control^49,50^.

As previously mentioned, Ad vectors are constructed by replacing genes involved in replication with genes of interest. Which replication genes are deleted impacts the immunogenicity of the vector, and past work dedicated to developing vectors for gene therapy purposes has focused on reducing the immunogenicity of the vector, culminating in the creation of helper-dependent or high-capacity Ads, which are completely devoid of viral genes and require a helper virus to be manufactured. However, for vaccine purposes the immune response created by the remaining viral proteins is a benefit, and many Ad vector vaccines therefore rely on E1/E3 deleted vectors, which strike a balance between packaging capacity, ease of production, and immunogenicity^53,54^.

## Engineering the adenovirus capsid

Adenoviruses were the first major viral vectors explored for gene delivery, and their structural biology has been comprehensively studied. This strong scientific understanding has resulted in the unique ability to engineer the Ad capsid proteins, enabling the development of complex engineered vectors with altered properties^55^.

**Fig. 1:**
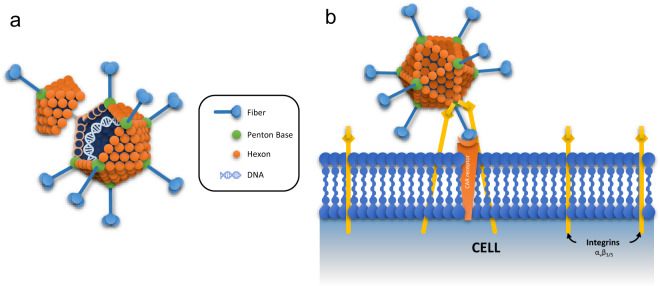
Adenovirus structure organization and interaction with host cell. **a** Adenovirus is a dsDNA, non-enveloped virus mainly composed by the structural protein, hexon, and other components associated with its interaction with the host cells (penton base and knobbed fiber). **b** The early stage of the infection cycle is marked by the knob domain of the viral fiber interaction with the Coxsackie and Adenovirus Receptor (CAR), followed by the penton-base with αvβ integrins present in the cell surface. CAR is the main receptor for the adenovirus serotype 5, however other serotypes utilize different receptors for cell entry.

The capsid is composed by three major proteins (Fig. 1a) with distinct structures and functions. The hexon is the most abundant protein of the capsid. It forms a pseudo-hexagonal structure that forms the 20 faces of the capsid and is held together by interactions with other capsid proteins^56^. The penton base is a pentameric protein that forms the capsid vertices and assists in virus internalization by interacting with integrins present on the host cell surface through the Arg-Gly-Asp (RGD) motif of the loop region^57,58^ (Fig. 1b). Lastly, the fiber is a trimeric protein containing a knob head region that primarily drives virus internalization through interactions with the target host cell receptor^50^ (Fig. 1a).

Each of these proteins has been engineered to alter the biological properties of the Ad vector, including tissue tropism, immunogenicity, and cell type specificity^55^. This capacity to alter the vector could result in next-generation vaccines with improved properties such as 1) the ability to avoid preexisting immunity after repeated vector doses^59^ 2) targeting to specific cell populations such as dendritic cells to potentially improve vaccine efficacy^60^ 3) increased thermostability for deployment to the third world^61^. However, as of the time of writing, all Ad vector vaccine candidates in development for COVID-19 are based on comparatively simple Ads, highlighting the differences between state-of-the-art research and the clinic. Success developing COVID-19 vaccines with these “first-generation” vectors will hopefully open the door to the translation of more complex and effective vectors in the future.

## Pre-existing immunity to adenovirus

Since their discovery, most scientific and clinical studies on Ads have focused on human adenovirus serotype 5 (Ad5), making it the best characterized and understood out of all Ad vectors. However, early discoveries indicated that the majority of the global population possesses pre-existing immunity to Ad5, likely generated through natural infection. This immunity was demonstrated to mitigate the ability of the vector to achieve gene delivery, impacting its ability to generate immune responses against the target antigen^62,63^. Pre-existing immunity to Ad5 was shown to decrease immunization potential in mice and non-human primate models^64,65^. Additionally, individuals enrolled in clinical trials for Ebola and HIV with prior exposure to human Ad5 had decreased specific T cell responses to the vaccine antigen^66–70^.

Nearly all components of the Ad particle including protein and nucleic acid are involved in the formation of anti-Ad immunity and can generate inflammatory responses through activation of toll-like receptor 2 (TLR2) and TLR9^71,72^, leading to the production of type I interferon and pro-inflammatory cytokines and chemokines^73,74^. Additionally, adaptive immune responses are triggered by epitopes present in Ad capsid proteins such as the hexon, fiber, and penton base. These interactions can induce CD4^+^ and CD8^+^ T-cell responses as well as the production of neutralizing antibodies. The presence of neutralizing antibodies and specific T cells against the adenovirus can prevent vectors of transducing the target cells and eliminate the transduced cells, respectively, inhibiting the vaccine efficacy^75,76^.

To overcome this issue, vectors from alternative Ad serotypes with low prevalence in the population have been developed. The human Ad35, Ad11, and Ad26, although less immunogenic than Ad5, are still effective vectors for vaccination purposes^65,77,78^. Additionally, Ads from other species such as chimpanzees, cattle, and pigs have been used as candidates for vaccine development^79–81^.

Despite the wide availability of different Ad vectors, the seroprevalence of the desired system must be carefully considered. Seroprevalence can differ across different regions globally, and cross-reactivity amongst different serotypes is also part of the equation: although neutralizing antibodies are usually serotype-specific, CD4^+^ cells specific to Ad5 can be cross-reactive with other serotypes such as Ad1, Ad3, and Ad35^82–85^. Additionally, the future of successful, widely distributed Ad vaccines such as the Oxford/AstraZeneca and Janssen/Johnson & Johnson COVID-19 vaccines remain unclear, as dosed individuals may develop inhibitory anti-vector immunity through the mechanisms detailed above. It remains to be seen if new vaccines based on the same vectors can be re-administered in the future, as may be required to control COVID-19 variants. The number of doses of the same vector which can be delivered without impacting efficacy and how much time these doses should be separated by remains unclear. These challenges have contributed to numerous public and private agencies investing significant research efforts in the development of a vast array of different Ad vectors for COVID-19, as detailed in the following sections of this review.

## Adenoviral vector vaccines for SARS-CoV-2

### Human adenovirus serotype 5 vaccines

#### Ad5-nCoV (Convidecia)

**Fig. 2:**
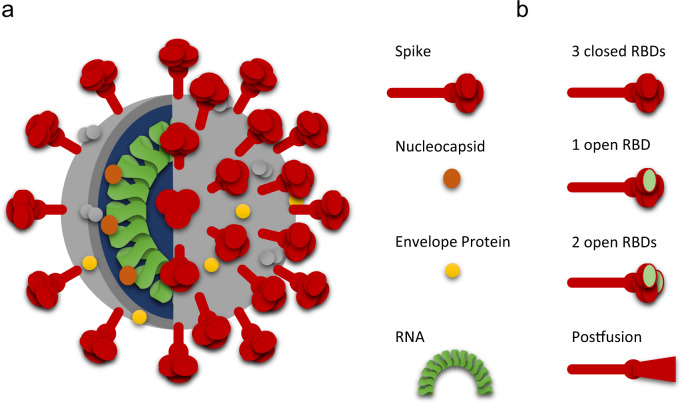
Coronavirus structure and relevant aspects for vaccine development. **a** Current vaccines are capitalizing in epitopes present in the SARS-CoV-2 proteins to elicit an immune responses. The major proteins used for vaccine development are the nucleocapsid, and the spike protein, essential for cell entry. **b** Spike protein can have conformation modifications protease-mediated. The stabilization of the protein in its prefusion form improves the protein expression as well as immunogenicity.

Ad5-nCoV (trade name: Convidecia) is a first-generation E1/E3-deleted Ad5 based vector carrying the full-length SARS-CoV-2 spike glycoprotein (Fig. 2a), developed in China by CanSino Biologics Inc. and the Beijing Institute of Biotechnology in early 2020. A summary of the preclinical data generated for this vector, as well as the others detailed in this review, can be found in Table 2. The efficacy of Ad5-nCoV was assessed in mice and ferrets, in which SARS-COV-2 replication occurs in the upper respiratory tract, but not in the lungs^86^. Both intranasal (IN) and intramuscular (IM) administration routes were tested, and IN resulted in complete protection against SARS-CoV-2 in the upper and lower respiratory tracts in mice. However, concerns regarding issues with IN administration in people with asthma led to IM being chosen for Ad5-nCoV vaccination in the first human clinical trials^86^.

**Table 2:** Comparative table of preclinical research studies with Adenoviral vector-based COVID-19 vaccine candidates.

Vaccine	Species (animal number per group)	Viral particle per animal	Regimen	Route of administration	Humoral immune response	Cellular immune response	Neutralizing antibodies	Viral RNA detected after vaccination	Prevents COVID-19 disease	Reference
Ad5-nCOV	Mus musculus (10)	5 × 10^7–9^	4 weeks before challenge	Intramuscular	Yes	Yes	Yes	No with mucosal vaccination	Yes	[86]
	Mustela putorious furo (6)	5 × 10^10^	4 weeks before challenge	Intranasal	Yes	Yes	Yes	No with mucosal vaccination	Yes	
hAd5 S-Fusion + N-ETSD	Mus musculus (5)	1 × 10^10^	Injections on Day 0, 21 and 28	Subcutaneous	Yes	Yes with CD4+ and CD8+ T-cell responses	Yes	NA	Yes	[97]
VXA-COV2-1	Mus musculus (6)	1 × 10^7^ 1 × 10^8^	Week 0 and Week 4	Intranasal	Yes	Yes with CD4+ and CD8+ T-cell responses	Yes	NA	Yes	[100]
AdCOVID	Mus musculus (10)	6 × 10^6^ 6 × 10^7^ 3.35 × 10^8^	Single	Intranasal	Yes with mucosal IgA	Yes with CD4+ and CD8+ T-cell responses	Yes	NA	Yes	[104]
Ad26.COV2-S	Rhesus macaques (4–6)	1 × 10^11^	Single dose	Intramuscular	Yes	Yes with CD4+ and CD8+ T-cell responses	Yes	No (only 1 animal in nasal swab)	Yes	[108]
Gam-COVID-Vac/ Sputnik V	NA	NA	NA	NA	NA	NA	NA	NA	NA	NA
GRAd-COV2	Cynomolgus macaques (4)	5 × 10^10^	Single-dose	Intramuscular	Yes	Yes with CD4+ and CD8+ T-cell responses	Yes	NA	NA	[120]
ChAdOX1-nCOV	Rhesus macaques (6)	2.5 × 10^10^	prime-only or a prime-boost regimen	Intramuscular	Only prime-boost regimen	Yes (did not increase after the second dose)	Yes	Only in nasal swab (not in the lungs)	Yes	[99]
ChAd-SARS-CoV-2	Mus musculus (5–9)	1 × 10^10^	Subsets with boost immunization at week 4	Intramuscular	Yes	Yes with CD4+ and CD8+ T-cell responses	Yes	Yes (low level in the lungs)	Yes	[138]
				Intranasal	Yes with mucosal IgA	Yes with CD4+ and CD8+ T-cell responses	Yes	No	Yes	
	Mesocricetus auratus (10)	1 × 10^10^	Single-dose	Intramuscular	Yes	NA	Yes	10-fold less*	Yes	[139]
				Intranasal	Yes	NA	Yes, several-fold higher than IM	100-fold less in nasal swab*	yes	

* Compared to ChAd-control immunized animals

*NA* not available, *IgA* immunoglobulin A, *IM* intramuscular, *Adm.* Administration, *IM i*ntramuscular, *IN* intranasal, *SC* subcutaneous, *N* nucleocapsid peptide, *S* spike, *Th1* 1 T helper cell, *NAb n*eutralizing antibodies, *IgG* immunoglobulin G, *IgA* immunoglobulin A, *BAL* bronchoalveolar lavage.

After a single dose IM administration in healthy individuals, both binding and neutralizing circulating antibodies were detected as well as antigen-specific CD4^+^ and CD8^+^ cells. The level of this response seems to be affected by preexisting immunity to Ad5 and advanced age^87,88^. Adverse events such as fever, fatigue, and muscle pain were observed in the population, but no serious events were reported. The phase 1 and 2 reports concluded that Ad5-nCoV is well tolerated and capable of inducing both humoral and cellular immunity. A comparative table of published clinical trials is presented in Table 3.

**Table 3:** Comparative table of clinical studies with Adenoviral vector-based COVID-19 vaccine candidates.

Developer	Name of Candidate Vaccine	Most Advanced Stage of Clinical Trial	Location of development	NCT Number of Corresponding Clinical Trial	Route of administration	Dose groups (viral particles)	Participants	RBD-specific ELISA Abs [AU]/mL	GMT of NAbs [AU]/mL	Interferon γ Responses (%)	Grade 1/2 adverse reactions (%)	Severe adverse reactions (grade 3) (%)
**CanSino Biologics Inc. and Beijing Institute of Biotechnology**	Ad5-nCOV	Phase 3	China	Phase 2 NCT04341389	Intramuscular Single dose	1 × 10^11^	253	656.5	19.5	90	73	9
						5 × 10^10^	129	571.0 (Day 28)	18.3 (Day 28)	88	74	1
						Placebo	126	-	-	-	-	-
**ImmunityBio, Inc. and NantKwest Inc**.	hAd5-S-Fusion+N-ETSD	Phase 1	USA	Phase 1b NCT04341389	Sublingual on Day 1 and 22	1 × 10^11^ or 5 × 10^10^	35	NA	NA	NA	NA	NA
**Vaxart**	VXA-COV2-1	Phase 1	USA	Phase 1 NCT04563702	Oral tablet on Day 1	1 × 10^10^ or 1 × 10^11^	35	NA	NA	NA	NA	NA
**Altimmune, Inc**.	AdCOVID	Phase 1	USA	Phase 1 NCT04679909	Intranasal spray, single or two doses	Low, Medium, or High	180	NA	NA	NA	NA	NA
**Janssen Vaccines & Prevention B.V. (Johnson & Johnson)**	Ad26.COV2-S	Phase 3	Netherlands	Phase1/2a NCT04436276	Intramuscular Single or two doses	1 × 10^11^	1045	478–586	224–354 (Day 29)	64	84	20
						5 × 10^10^		625–788 (Day 29)	827–1266 (after 2 doses)	51	65	9
						Placebo		–	–	–	23–26	0
**Gamaleya Research Institute**	Gam-COVID-Vac/ SputnikV	Phase 3	Russia	Phase 3 NCT04530396	Intramuscular Prime (rAd26-s)-boost (rAd5-S) on Day 0 and Day 21	1 × 10^11^	21977	8996 (Day 42)	44.5 (Day 42)	NA	94	0.3–0.4
**ReiThera/LEUKOCARE/Univercells**	GRAd-COV2	Phase 2/3	Italy	Phase 1 NCT04528641	Intramuscular Single dose	5 × 10^10^, × 10^11^ or 2 × 10^11^	90	NA	NA	NA	NA	NA
**University of Oxford/ AstraZeneca**	ChAdOX1-nCoV	Phase 4	England	Phase 2/3 NCT04400838	Intramuscular: Single or two doses	5 × 10^10^(prime), 2.2 × 10^10^ (boost)	12390	9705–20713 (Day 28)	144–193 (Day 42)	100	61–88	0–4
**Gristone Oncology**	ChAd68-S and ChAd68-S-TCE	Phase 1	USA	Phase 1 NCT04776317	Intramuscular: Two or three doses	5 × 10^10^ or 1 × 10^11^	130	NA	NA	NA	NA	NA
**Bharat Biotech**	BBV154	Phase 1	USA, India	Phase 1 NCT04751682	Intranasal, single or two doses	1 × 10^10^	175	NA	NA	NA	NA	NA

Numbers in parentheses refer to the day post-vaccination when antibodies were measured.

*VP* virus particles per participant, *NCT* national clinical trial, *NA* not available, *RBD-specific ELISA Abs* Receptor Binding Domain-specific Antibodies measured by Enzyme linked Immunosorbent Assay (ELISA), *GMT of NAbs* Geometric Mean Neutralizing-Antibody Titer against live SARS-CoV-2, %= percentage of the study participants, *[AU]/mL* arbitrary unit/milliliter.

To assess the efficacy of Ad5-nCoV, a multicenter, double-blind, placebo-controlled phase 3 clinical trial is ongoing (NCT04526990). Still in the recruitment phase, the study is reported to have started in September 2020 and is estimated to be completed in January 2022. In February 2021, an interim analysis reported that Ad5-nCOV prevented 65.28% of symptomatic cases and 90.07% of severe diseases. Both analyses were carried out after a single dose^89^, and resulted in Ad5-nCoV being approved for use in China, Hungary, Mexico, and Pakistan^90–93^.

#### hAd5-S-Fusion+N-ETSD vaccine

As previously discussed, the development of potentially more virulent SARS-CoV-2 variants has driven efforts to develop novel vaccines targeted towards them. In particular, the D614G spike protein mutation has raised concerns regarding the potential resurgence of a virus variant refractory to the immunity elicited by the current vaccines still in development^94,95^. The hAd5-S-Fusion+ETSD vaccine, developed by ImmunityBio, Inc. and NantKwest Inc. (USA), was therefore designed to deliver both the S-Fusion and N-ETSD proteins, which are engineered versions of the SARS-CoV-2 spike and nucleocapsid proteins, respectively. The nucleocapsid protein has been shown to be conserved across SARS-CoV-2 variants, potentially enabling vaccines carrying this antigen to maintain protection against new and emerging variants^96^. The vector itself is based on a second-generation Ad with the E1, E2b, and E3 genes deleted that has been previously used in the presence of pre-existing immunity against Ad5. Pre-clinical data showed that immunization in a murine model elicited humoral and cellular immune responses against both spike and nucleocapsid proteins of the SARS-COV-2 virus^97^. Optimization of the nucleocapsid antigen for lysosomal/endosomal localization enhanced CD4+ T cell responses, suggesting that the bivalent vaccine might provide long-lasting protection against SARS-CoV-2 and spike protein variants.

The hAd5-S-Fusion+N-ETSD vaccine is currently in phase 1 clinical trial to assess the safety, reactogenicity, and immunogenicity of the vaccine in various doses administered either subcutaneously or orally (NCT04591717, NCT04732468). The study is currently recruiting healthy individuals and is expected to be completed in late 2021. In March 2021, ImmunityBio, Inc. announced positive interim phase I safety data of their vaccine candidate in both formulations. No serious adverse events were reported after six participants were administered subcutaneously with the vaccine. The oral capsule formulation of this vaccine candidate would be advantageous to facilitate cold chain-free vaccine distribution, as it can be stored at room temperature.

#### Vaxart

Most of the vaccines currently in development are administered via the IM route. Although systemic immunization by IM vaccines confers protection that prevents disease establishment and progression, alternate routes of delivery may possess unique advantages. Vaxart’s vaccine platform uses oral tablets to administer recombinant Ad vectors expressing the full-length SARS-CoV-2 spike protein, the nucleocapsid protein, and a Toll-Like Receptor-3 (TLR-3) agonist as an adjuvant. This technology was shown in a phase 2 trial for influenza to be well tolerated and generate protective immunity^98^. The authors hope that induction of both mucosal and systemic immunity via their oral tablet platform will provide the means to circumvent barriers related to the broad distribution of vaccines to regions with limited cold chain infrastructure as well as reduce logistic costs. Additionally, it was concluded that stabilizing the spike protein in its pre-fusion conformation is not necessary in nucleic acid vaccines, since it does not induce higher neutralizing antibody (NAb) titers in murine models compared to the non-stabilized version. Nevertheless, this has not been reported in other Ad vaccines^99,100^.

Vaxart is currently carrying out a phase 1 clinical trial (NCT04563702) to assess safety and immunogenicity. Based on preliminary data, a press release by Vaxart stated that VXA-CoV2-1 is well-tolerated and capable of eliciting strong CD8+ T-cell responses^101^. Most recently, the organization analyzed data from the Phase I trial against similar data from volunteers treated with Pfizer or Moderna’s vaccines and claimed that the Vaxart vaccine produced larger CD8+ T cell responses^102^.

#### AdCOVID

Another way to induce mucosal and systemic immunity is IN vaccination. AdCOVID, developed by Altimmune, Inc., is an Ad5 vector encoding the receptor-binding domain (RBD) of the spike protein that showed encouraging immunogenicity results after a single IN administration in mice. The observed mucosal and systemic antigen-specific CD4+ and CD8+ T cell responses were characterized by a T-helper 1 (Th1) type cytokine profile. Importantly, Th1/Th2 balance has been associated with COVID-19 severity - Th1 coordinated immune responses have been linked to positive prognoses, while high Th2 responses may lead to worse prognoses^103^. Additionally, AdCOVID was capable of inducing slightly higher antibody responses compared to vaccines expressing the full length or S1/S2 domain of the spike^104^. AdCOVID is expected to be tested in a double-blind, randomized, placebo-controlled human phase 1 study starting in February 2021 (NCT04679909).

## Human adenovirus serotype 26 vaccines

### Janssen/Johnson & Johnson (Ad26.COV2-S)

The Ad26.COV2-S vaccine developed by Janssen Vaccines & Prevention B.V. (Johnson & Johnson) uses a first-generation Ad26 vector (E1/E3 deleted) to deliver the pre-fusion stabilized SARS-CoV-2 spike protein. This protein has been stabilized through a mutation in a furin cleavage site, and a proline substitution. Details of these modifications are reviewed in Bos et al., 2020^105^.

Pre-clinical data on this vector showed that delivery of the stabilized spike triggers strong humoral and cellular Th1 interferon-gamma (IFN-γ) immune responses with the presence of both neutralizing and RBD binding antibodies^105,106^. The same research group assessed the Ad26.COV2-S immunization efficacy in Syrian hamster model (*Mesocricetus auratus*). This animal model is naturally susceptible to SARS-CoV-2 infection and develops mild-to-severe disease with symptoms similar to human COVID-19 disease^106,107^. A single-dose administration of the vector protected the animals against severe SARS-CoV-2 pneumonia and mortality^106^. In a non-human primate model, the vaccine elicited strong neutralizing antibody production after a single dose intramuscular administration and conferred protection against the SARS-CoV-2 challenge. The authors noted that additional studies are needed to assess the mucosal delivery of this vector, and to evaluate the durability of the established near-complete protection against SARS-CoV-2 infection^108^.

A phase 1/2a clinical trial utilizing the vector is currently ongoing. Interim analysis of the double-blind, randomized, placebo-controlled study revealed that the vaccine candidate elicited humoral and cellular immune responses in both young and elderly participants after a single dose. Regarding safety, most of the adverse events were mild to moderate and the reactogenicity was concluded to be dose dependent^109^. The efficacy and safety of low dose (5 × 10^10^ viral particles) Ad26.COV2-S are now being assessed in two multicenter phase 3 clinical trials, either in a single dose or two-dose regimen (NCT04505722 and NCT04614948, respectively) and their completion is estimated in March 2023.

In January 2021 Janssen released an interim report with efficacy data from 44,325 individuals vaccinated in Argentina, Brazil, Chile, Colombia, Mexico, Peru, South Africa, and the United States. The analysis showed the vaccine to be 66% effective at preventing moderate to severe COVID-19 cases 28 days past immunization and 85% effective at preventing the severe disease^110^. More recent data for the clinical trial was generated in South Africa and Brazil, where SARS-CoV-2 variants (respectively 501Y.V2 and P1) were broadly circulating, which may have influenced the efficacy data^111^.

The Ad26.COV2-S single-shot vaccine has been approved for emergency use in the US and another 40 countries in individuals 18 years of age and older^112^. However, in mid-April US regulators temporarily paused Ad26.COV2-S vaccine administration to investigate 15 reported cases of severe thrombosis with thrombocytopenia, out of 7.98 million doses administered. Similar events have been reported in individuals receiving the ChAdOx1-nCov19 vaccine outside the US. Following an FDA/CDC review and a risk/benefit analysis, vaccine administration was resumed - the risk of developing the rare vaccine-induced condition, termed as thrombosis with thrombocytopenia syndrome (TTS), is very low, and the risk/benefit strongly favors vaccination^113,114^. A more detailed consideration of this issue is provided in future sections of this review.

### Sputnik V (gam-COVID-vac)

Sputnik V is a vaccine candidate developed by the Gamaleya Research Institute in Russia. The vaccination protocol consists of a two-dose regimen utilizing two human Ads: Ad26 as prime and Ad5 as a boost. Heterologous Ad vector prime-boost immunization protocols, where a different type of virus is applied at each dose, are used as a strategy to circumvent immune responses against the viral vector^115,116^. Additionally, heterologous prime-boost regimens have been shown to be more immunogenic than homologous prime-boost regimens^115^. From a technical standpoint, both Ads carry the same DNA encoding the full-length SARS-CoV-2 spike protein sequence, with no further modifications.

Two non-randomized phase 1/2 trials were conducted to evaluate the safety and immunogenicity of the vector individually as well as in a prime-boost protocol using either a frozen (NCT04436471) or lyophilized (NCT04437875) vaccine formulation. The trials found both formulations safe and well-tolerated with no reported serious adverse events. Regarding immune responses, all enrolled individuals had increased levels of circulating neutralizing antibodies against the SARS-CoV-2 spike protein. Cell-mediated immune responses were also elicited in all subjects by both formulations^117^.

To assess the efficacy of Sputnik V, two phases 3 clinical trials are being carried out (NCT04530396, NCT04564716). In an interim report of the NCT04530396 trial, involving 21,977 adults, the vaccine candidate with a prime-boost regimen showed 91.6% efficacy against COVID-19. Regarding safety, the reported adverse events were mostly graded 1 and none of the reported serious adverse events could be associated with the vaccination^118^. As of April 2021, Sputnik V was approved for emergency use in Russia and several other countries^119^.

## Non-human adenovirus vaccines

### ReiThera/LEUKOCARE/Univercells (GRAd-COV2)

The GRAd-COV2 vaccine in development by ReiThera/LEUKOCARE/Univercells uses a Gorilla Adenovirus, GRAd32 to deliver an engineered SARS-CoV-2 spike protein sequence. This virus belongs to the group C adenovirus family, similar to human Ad5 and ChAd3^120^, and the spike protein modifications aimed to increase the pre-fusion protein form stability to improve its expression as well as its immunogenicity^121^. The vector is E1 and E3 deleted, and the E4 coding region was swapped with human Ad5 ORF6 to improve vector manufacturing yield.

In a pre-clinical study in mice, the vector elicited strong humoral and cellular immune responses with increased titers of circulating NAbs and antigen-specific T cells. The results were similar in a nonhuman primate model - GRAd-COV2 elicited the production of neutralizing antibodies and high levels of primed CD8^+^ and CD4^+^ cells. Single-dose administration of the vector is an important benefit compared to two-dose vaccination, especially from a practical point of view. The authors speculate that species C Ad vectors may be the most potent Ad vaccines, potentially informing future research into novel vectors. However, this must be confirmed in clinical settings^120^.

To assess the safety and immunogenicity of the GRAd-COV2 vaccine a phase 1 open-label, a dose-escalation clinical trial is ongoing (NCT04528641). An interim analysis of the first cohort of the study (individuals 18-55 years) revealed that the vaccine-induced specific humoral and cellular responses to the SARS-CoV-2 S protein without triggering severe adverse events^122^. More recently Reithera announced a phase 2/3 clinical trial (NCT04791423) to assess dosing and efficacy. The study started in March 2021 and is expected to be completed in April 2022.

### Oxford/AstraZeneca (ChAdOX1-nCoV)

During the 2012 Middle East respiratory syndrome coronavirus (MERS-CoV) outbreak, the Oxford group developed a vaccine using their ChAd (chimpanzee Ad) technology, which circumvents pre-existing immunity to Ad5. The vector was developed from an Ad isolated from a chimpanzee fecal sample, and vectorized by deletion of E1/E3 and modifications in E4 (*E4Orf4*, *Orf6,* and *Orf6/7* swapped with human Ad5)^123^. With the SARS-CoV-2 outbreak, the same Ad backbone was used to create ChAdOX1-nCoV, encoding the SARS-CoV-2 spike protein^124^. The already well-established protocols for vector construction and assessment uniquely positioned the research group ahead of the pack in the SARS-CoV-2 vaccine development race.

In murine and non-human primate pre-clinical models ChAdOX1-nCoV was shown to be a promising vaccine candidate^99^. After one IM dose, the vaccinated mice showed increased levels of NAbs specific to the spike protein. T cell responses were also elicited against the spike protein, and the overall response was Th1 oriented. The same features were observed in a study with rhesus macaques, which showed reduced SARS-CoV-2 viral load in the bronchoalveolar lavage, as well as reduced pneumonia development^99^.

In a phase 1/2 single-blind, randomized controlled trial using a prime-boost regimen, ChAdOX1-nCoV showed a safe profile for human application, with no serious adverse events observed (NCT04568811). Circulating SARS-CoV-2 specific T-cells were found in the vaccinated subjects as well as increased levels of NAbs in all individuals^125^. After completion of this trial, ChAdOX1-nCoV safety and immunogenicity were evaluated through a single-blind, randomized placebo-controlled phase 2/3 clinical trial study (NCT04400838). The immunogenicity was tested in different age groups (18–55, 56–69, ≥ 70 years), and was reported to be comparable in all groups after the second dose of ChAdOX1-nCoV. Primary efficacy data from an ongoing phase 3 was published in December 2020, which showed approximately 75% overall efficacy of ChAdOX1-nCoV in preventing symptomatic COVID-19. Moreover, longer intervals (> 12 weeks) between prime and boost doses were associated with higher antibody response. The possibility of a delayed administration of the booster dose gives time to manufacture additional doses while the first dose is being distributed^126^. The study completion is expected in October 2022 (NCT04324606, NCT04400838, and NCT04444674).

Following these studies, the vaccine obtained full approval in Brazil, and emergency use authorizations in numerous other countries, including the European Union^127^. Additionally, recently published data from a Phase 3 trial in the US confirmed the efficacy of the vaccine^128^. The success of the vaccine has led the researchers to initiate trials with new vectors targeted towards new SARS-CoV-2 variants, as well as a nasal spray^129,130^. However, in mid-March several countries paused their vaccination programs due to reports that the vaccine could potentially cause dangerous blood clots in certain individuals, similar to the Johnson & Johnson vaccine^131^. Although major regulatory agencies confirmed the safety of the vaccine, these rare clotting events remain a significant concern delaying administration of the vaccine, with additional countries pausing rollout in some populations^132–135^.

### Bharat biotech (ChAd-SARS-CoV-2/ BBV154)

ChAd-SARS-CoV-2 is a chimpanzee adenoviral (ChAd36) vector containing the sequence for the SARS-CoV-2 spike protein stabilized in the prefusion form by two proline substitutions (K986 and V987)^121,136^. Similar to other simian Ads, the seroprevalence of the vector in the human population is very low^123,137^.

In a murine model for SARS-CoV-2 infection, intramuscular administration of the vaccine-induced humoral and cell-mediated responses, with high levels of Nabs and CD8^+^ T cell responses conferring SARS-CoV-2 immunity. When administered intranasally, the vaccine also induced mucosal immunity and resulted in sterilizing immunity, preventing respiratory tract infection and inflammation. Intranasal vaccination of this vector is a suitable platform for the prevention of SARS-CoV-2 infection and transmission in humans, but further studies are required to evaluate how long the protective immunity lasts^49,138^.

Additionally, the same vaccine candidate was tested in a Syrian hamster model. The researchers compared the efficacy of IM vs. IN delivered ChAd-SARS-CoV-2 vectors and found IN delivery resulted in 6-fold higher neutralizing antibody titers compared to IM delivery of the vaccine candidate. Moreover, reduced infectious viral RNA was observed in the lungs and nasal cavity upon SARS-CoV-2 challenge only after IN immunization. This head-to-head comparison of IM vs IN delivery of the same vaccine vector highlights the importance of the immunization route and supports IN administration to achieve sterilizing immunity against SARS-CoV-2^139^.

With promising results, ChAd-SARS-CoV-2 (now named as BBV154) is currently in phase 1 clinical trial (NCT04751682) carried out by Bharat Biotech International Limited, in India. The study aims to evaluate the immunogenicity and reactogenicity in healthy adults and establish the safety profile of the vaccine in one and two-dose regimens. The trial is active but has ceased recruitment, and is expected to be completed in November 2021.

### ChAdV68-S and ChAdV68-S-TCE

Gritstone Oncology’s COVID-19 vaccine platform aims to elicit strong CD8^+^ T cell and antibody responses by a combination of two potent vaccine vectors: a Chimpanzee adenovirus serotype 68 (ChAdV68) and a self-amplifying mRNA (SAM) expressing either the SARS-CoV-2 spike protein alone, or the spike plus additional SARS-CoV-2 T-cell epitopes. Using additional T-cell epitopes from the nucleoprotein and other gene regions which have been identified through studies of COVID-19 patients has the potential to maximize cellular and humoral immune responses and to protect against new spike mutant strains-patients who recovered from SARS have long-lasting memory T-cell responses against conserved proteins such as the nucleoprotein^140^. The advantage of SAM vectors over conventional non-amplifying mRNA vectors is the potential application of a lower dose, as SAM vectors can replicate the mRNA payload, essentially amplifying the dose delivered in vivo. This potentially reduces the manufacturing cost and time, enabling faster vaccination of a greater number of people^141^.

A phase 1 study (NCT04776317) of this second-generation COVID-19 vaccine has been started. The dose-escalation trial compares heterologous ChAdV68 prime/SAM boost and homologous SAM prime/SAM boost regimens within different patient groups. Gritstone Oncology believes that its heterologous vaccine platform is well-suited to become a pan-coronavirus vaccine in the future.

## Vaccine induced thrombocytopenia (VIT)

In March and April 2021, reports of rare blood clots linked to the Oxford/AstraZeneca and Johnson & Johnson COVID-19 vaccines began to circulate^142,143^. In several countries administration of each vaccine was paused, then restarted after reviewing of the data, with the exception of Denmark, where administration of the Oxford/AstraZeneca vaccine remains on hold. Although little is known so far, these events have been characterized as being similar to heparin-induced thrombocytopenia (HIT), another rare blood clotting disorder. The events also seem to be specific to Ad vaccines, with links being established only with the Oxford/AstraZeneca and Johnson & Johnson vaccines. However, one finding that did emerge was the near ubiquitous presence of anti-platelet factor 4 (PF4) antibodies across patients suffering from VIT^144^. Platelet factor 4 is able to complex with negatively charged polymers in the blood and has been strongly implicated in HIT^145^. Briefly, PF4 can bind to heparin and other similar structures, which in some individuals can create an autoimmune reaction against the complexes. This causes a series of downstream reactions which ultimately lead to thrombocytopenia^145^.

Since this initial discovery, a variety of hypotheses have been put forward to attempt to explain the links between VIT, PF4, and Ad vaccines. An early study demonstrated that anti-PF4 antibodies and the SARS-CoV-2 spike do not bind each other, likely eliminating the possibility that the vaccine antigen itself is responsible for VIT^146^. This is in alignment with the fact that VIT seems to only occur in Ad vector COVID-19 vaccines.

Alternatively, Ads have been shown to activate platelets in the bloodstream and induce thrombocytopenia, potentially causing a release of PF4 and leading to the induction of anti-PF4 antibodies^147,148^. If Ads are able to enter the bloodstream, either through inappropriate vaccine administration or other mechanisms, this hypothesis could be quite convincing. Building on this idea, one study found that the Oxford/AstraZeneca vaccine could bind with PF4 and induce pro-inflammatory immune responses at the site of injection^149^. Additionally, two studies have found significant levels of protein-based impurities in the Oxford/AstraZeneca vaccine, including heat-shock proteins^149,150^. Such impurities may also contribute to immunological reactions to the vaccine and may help explain differences in VIT rates between the major approved Ad vaccines, assuming each vaccine uses a slightly different purification process. Importantly, many of the publications studying this issue are currently only available as pre-prints and have not yet been peer-reviewed.

Numerous questions regarding Ad vaccine VIT remain unanswered. If the critical initial step in generating VIT is entry of the Ad particle or other vaccine components into the bloodstream, might an IN or oral administration route solve the problem? Alternatively, it has been posited that endothelial cells transduced via the vector could express the SARS-CoV-2 spike protein into the bloodstream and create an anti-PF4 immune reaction through interactions between the spike, damaged endothelial cells, and platelets^151^. If this hypothesis is correct, why doesn’t VIT also occur with mRNA vaccines? Historically, Ad vectors have been selected for vaccines on the basis of their ability to generate strong immune responses, essentially acting as a type of adjuvant to the actual antigen. Might less-immunogenic Ads help circumvent the VIT issue, if they can maintain strong anti-transgene immune responses?

Thankfully, VIT seems to remain a rare issue, and clinical management of the disease has improved rapidly, with successful treatment of one individual reported^151^. Hopefully, with increased awareness and proper treatment options any further tragic deaths can be avoided. However, a continued study into this issue is critical, and characterization of new administration routes for Ad vaccines and potentially new vectors will hopefully lead to complete elimination of VIT.

## Ad vaccines: achievements and future perspectives

The rapid development of a vaccine has historically been extremely challenging, but as of May 2021, there are 100 COVID-19 candidate vaccines currently in clinical trials^152^. The ongoing pandemic and the need for fast development of vaccines have paved the way for the use of novel technologies such as Ad vectors and mRNA at a global scale-as of May 2021, ten different Ad vector vaccines are under clinical investigation and four are approved for emergency use (ChAdOX1-nCoV by Oxford/AstraZeneca, Ad26.COV2-S by Janssen/Johnson & Johnson, Gam-COVID-Vac/Sputnik V by the Gamaleya Research Institute, and Ad5-nCOV by CanSino Biological Inc.). These vaccines employ human (Ad5 and Ad26) and chimpanzee serotypes to deliver the coding sequence for SARS-CoV-2 peptides. Two of them, developed by Janssen/Johnson & Johnson and Oxford/AstraZeneca, have been associated with extremely rare incidences of vaccine-induced thrombotic thrombocytopenia, but both vaccines are considered highly efficacious against COVID-19 severe disease and remain recommended by regulators.

Despite the positive immunogenicity and safety results of the current Ad vaccines, more time is necessary to assess for how long immunity lasts and address the challenge of rare adverse events. Employing Ads with refined technology could allow these barriers to be traversed. Genetic modification has been used to modulate the interaction of Ads with the immune system, including the creation of cell-specific vectors to enhance immunization,^153–155^ and hexon modifications to mitigate vector inactivation from pre-existing immunity^156^. The capacity of Ad to undergo such complex engineering could hopefully lead to the development of new vectors which 1) evade pre-existing or induced immunity, 2) induce a strong, long-lasting, cross-reactive immune response after a single dose 3) are safe to administer to the global population without risk of rare side effects and 4) are shelf-stable, cheap, highly scalable and capable of distribution to both first and third-world countries.

Additionally, the two EUA approved mRNA COVID-19 vaccines developed by Moderna and Pfizer-BioNTech and the Ad vector vaccine candidates (Ad5-nCoV, ChAdOx1-nCoV, Gam-COVID-Vac, Ad26.CoV2-S) advanced to Phase 3 clinical trials are administered via intramuscular injections that confer protection against SARS-CoV-2 infection in the lungs, but cannot provide sterilizing mucosal immunity^88,109,117,157–159^. In contrast, Ad vector COVID-19 vaccine candidates (VXA-CoV2-1, AdCOVID, ChAd-SARS-CoV-2) administered via intranasal route have achieved sterilizing immunity in preclinical animal models^86,104,138^. The potential advantages of an intranasally administered Ad vector vaccine are the ability to block transmission of the infectious agent^160^, bypass preexisting immunity to the vector^161^ and stimulate strong humoral and cellular immunity at both local and systemic levels^161^, all with a non-invasive and easy administration^162^. Exploration of alternate dosing routes may therefore help achieve more effective protection and enable vaccines in other pharmaceutical forms with a more robust shelf-life.

## Conclusions

The emergency posed by the SARS-CoV-2 pandemic has shown that Ad vectors are strong vaccine candidates. Clinical trials with Ad vaccines have demonstrated they are safe in humans, with no serious adverse events observed in the vast majority of individuals. These same trials have shown the capacity of Ad vaccines to produce strong protective humoral and cellular immune responses, even after a single dose in some cases. These studies have demonstrated Ad vectors are amongst the most promising vaccine platforms in the race against the SARS-CoV-2 pandemic.

The SARS-CoV-2 pandemic has had a global impact unprecedented in the twenty-first century, with millions of lives lost and a tremendous strain on the economy. A small silver lining to this tragedy has been the incredible advancements in vaccine technology and the increased cultural awareness of their importance in public health. Although this breathtaking pace of development will undoubtedly slow without an active pandemic to drive it, it is the authors’ hope that the development of highly efficacious and safe vaccines capable of rapid deployment will continue, in order to prepare the world for the next pandemic.

## Data Availability

No data was generated and/or analyzed for the review article.
